# MicroRNAs may provide new strategies in the treatment and diagnosis of diabetic retinopathy: Importance of VEGF

**DOI:** 10.22038/ijbms.2021.52164.11807

**Published:** 2021-03

**Authors:** Vahid Akbari Kordkheyli, Mohammad Amir Mishan, Abbas Khonakdar Tarsi, Abdolkarim Mahrooz, Mozhgan Rezaei Kanavi, Ali Hafezi-Moghadam, Abouzar Bagheri

**Affiliations:** 1 Department of Clinical Biochemistry and Medical Genetics, Student Research Committee, Faculty of Medicine, Mazandaran University of Medical Sciences, Sari, Iran; 2 Ocular Tissue Engineering Research Center, Research Institute for Ophthalmology and Vision Science, Shahid Beheshti University of Medical Sciences, Tehran, Iran; 3 Department of Clinical Biochemistry and Medical Genetics, Molecular and Cell Biology Research Center, Faculty of Medicine, Mazandaran University of Medical Sciences, Sari, Iran; 4 Molecular Biomarkers Nano-Imaging Laboratory, Brigham and Women’s Hospital, Boston, Massachusetts, USA; 5 Department of Radiology, Harvard Medical School, Boston, Massachusetts, USA

**Keywords:** Diabetic retinopathy, Gene regulation, Molecular targeting, miRNAs, VEGF

## Abstract

Diabetic retinopathy (DR) is ocular microvascular complications of diabetes mellitus. Along with the increasing prevalence of diabetes worldwide, DR has come into the major cause of human blindness. Several studies have demonstrated the important roles of the expression alteration in the proteins contributed to vascular dysfunction during DR, especially vascular endothelial growth factor (VEGF). However, there is a need for further mechanistic research in this context to design new therapeutic and diagnostic programs. MicroRNAs (miRNAs, miRs) have been introduced as key controllers of gene expression in a variety of biological processes including differentiation, proliferation, and metabolism. Altered expression of miRNAs during DR development indicates a close relationship between these regulatory molecules and DR through regulating gene expressions. This review discusses and updates the functions of miRNA-dependent pathways and key roles of VEGF in the DR, which may increase our understanding and ability to target these small but important molecules to efficiently improve therapeutic and diagnostic approaches.

## Introduction

Diabetes mellitus (DM) is associated with various blood vessel abnormalities in different tissues, particularly the eye that is known as diabetic retinopathy (DR). Along with the global prevalence of DM, DR as the main consequence of diabetes-related microvascular dysfunction in the eye has been coming one of the commonest causes of human blindness worldwide (1). Based on the reports, DR causes 2.6% of all cases of blindness worldwide. Globally, 93 million patients with DR and 28 million patients with vision-threatening DR have been reported, introducing DR as a serious health challenge in the world (2). DR may be proliferative (PDR) or non-proliferative (NPDR). In both types, there is a risk of diabetic macular edema (DME) that can trigger visual impairment. PDR, unlike NPDR, is more progressive and is associated with the formation of a proliferative neovascular tissue at the inner aspect of the retina, optic disc, or iris that can lead to retinal hemorrhages, exudates, and finally blindness. Mild and moderate NPDR is not vision-threatening, does not always develop to proliferative condition, and may regress if glycemia is controlled. For vision-threatening DR, there are confined treatment options. Laser therapy or pan-retinal photocoagulation is effective at inhibiting further visual impairments. Without treatment, most of the DR patients may lose their vision in 5-10 years after diagnosis (3). Therefore, early diagnosis of the DR is vital to slow its progression and to treat it. The severity of DR is associated with duration of diabetes, hyperglycemia, proteinuria, and hypertension; so, precise control of excess blood sugar and hypertension are the most important ways to significantly reduce the risk of poor prognosis in DR. However, in some patients, the vascular complications of DR progress are independent of blood sugar values that are known as metabolic memory (4).

Several studies have demonstrated the importance of changes in the expression of proteins contributed to vascular dysfunction during DR, such as pigment epithelium-derived factor (PEDF), angiopoietin (Ang), insulin-like growth factor (IGF), bone morphogenetic protein (BMP), and vascular endothelial growth factor (VEGF) (5-7). Normal expression of VEGF in the retinal pigment epithelial cells (RPECs) is necessary for preserving the structural and functional homeostasis, but its Over-expression has a crucial role in the pathogenesis of conditions such as oxidative stress, ischemia, inflammatory response, and hyperglycemia through increasing vascular permeability and pathological angiogenesis (8, 9). It was revealed that different miRNAs can regulate *VEGF* expression in DR (10). However, the exact mechanism controlling the processes has not been well understood, and there is a need for further clinical and mechanistic research in this context to help design new therapeutic and diagnostic approaches.

MicroRNAs (miRNAs, miRs) as the key regulators of the gene expression are involved in controlling various cellular processes, and DNA methylation and histone acetylation are known as epigenetic regulatory mechanisms of the gene expression (11-13). In recent years, the significant role of miRNAs in the pathogenesis and progression of DR through regulating gene expression has been demonstrated in many studies (Table 1). This review addresses the roles of miRNAs and their targeting pathways in DR, which may increase our understanding and ability to target these important molecules and may help to achieve further clinical benefits.

***Epidemiology of diabetic retinopathy***

There is no exact and updated knowledge about the global prevalence of DR, particularly vision-threatening phases, including PDR and DME, but analysis of 35 worldwide studies on diabetic patients revealed that the overall age-standardized prevalence of DR was 34.6%, PDR was 6.96%, DME was 6.81%, and vision-threatening diabetic retinopathy (VTDR) was 10.2%. It can be estimated that 92.6 million adults had DR, 17.2 million had PDR, 20.6 million had DME, and 28.4 million had VTDR. There was no discernible sex difference in the prevalence of DR and DME. The incidence of DR subgroups varied among ethnic groups and was highest among African Americans and lowest among Asians. The prevalence of DR was higher among people with type 1 than type 2 diabetes (77.3 versus 25.2%). It is estimated that the global prevalence of diabetes will be increased from 415 million in 2015 to 642 million patients in 2040 (4, 14). Lamparter and colleagues illustrated a prevalence of 8.2% for DR among the pre-diabetic patients in Mid-Western Germany (7.2% had mild NPDR, 0.4% moderate NPDR, and 0.2% had severe NPDR) (15). The statistics are in line with those from Shanghai where the prevalence of DR was 8% among pre-diabetics populations (16).

***Pathophysiology of*** ***diabetic retinopathy and VEGF role***

Precise mechanisms of DR are uncertain, but it seems that hyperglycemia is the key element underpinning the pathophysiology of DR. Major biochemical pathways in the development of hyperglycemic retinopathy have been identified, which includes accumulation of advanced glycation end-products (AGEs), and activation of polyol, hexosamine, protein kinase C, and polymerase (ADP-ribose) pathways (17, 18). Also, inflammation, oxidative stress, microcirculation failure, and mitochondrial damage that increases inflammatory mediators, transcription factors, chemokines, and surface adhesion molecules, can damage the blood-retina barrier (BRB) and increase VEGF and hormones, causing DME and PDR (19). It has been demonstrated that dysfunction and deterioration of retinal neuron precedes overt microcirculatory impairments in the early steps of DR and can reduce contrast sensitivity and color perception, limits the visual field, and induces an abnormal dark adaption (20, 21). Hyperglycemia-induced reactive oxygen species (ROS) accumulation in the mitochondrial electron transportation chain stimulates oxidative stress, which is a fundamental process for diabetic complications. These ROS could damage mitochondrial DNA and further linked to VEGF overexpression, and thus enhanced retinal endothelial cell (EC) proliferation. AGEs protein stimulates the intracellular generation of ROS by NADPH oxidase, and is related to the expression of VEGF and subsequently retinal neovascularization (22, 23). Current evidence suggests that inflammatory mediators such as monocyte chemoattractant protein-1, tumor necrosis factor alpha (TNFα), Interleukin-1 (IL-1), intercellular adhesion molecule-1 (ICAM-1), C-reactive protein (CRP), and vascular adhesion molecule-1 (VCAM-1) released by injured cells, inflammatory and microglial cells in the retina induce chronic subclinical inflammation that is responsible for many of the signature vascular lesions and pathogenesis of DR. These inflammatory cytokines induce oxidative stress, leukostasis, and microthrombosis in the diabetic retina, which can result in the retinal ischemia in DR, neuronal injuries, neuronal death, and inadequate perfusion of tissue, triggering the release of growth factors that increase neovascularization, a characteristic of PDR (24). Most previous studies focused on microcirculation (ECs and pericytes) damages in the pathogenesis of DR (25). A spectrum of retinal circulation impairments that occur during DR includes the breakdown of the inner BRB, microaneurysms, intraretinal hemorrhages, intraretinal increase of extravasated lipids and proteins, increased vascular permeability, focal venous dilatation, aberrant communications between retinal arterioles and venules, and dysregulated angiogenesis. These events cause the macromolecules leakage from blood vascular into the interstitial places of the retina. Further, changes in pericytes and glial cells in the capillary membrane induce leakage from the inner BRB (26, 27). These changes along with VEGF releasing result in vascular hyperpermeability and accumulation of fluid in the retina, which appears as DME when it involves the macula, the area subserving central vision (28).

An increment level of VEGF has been indicated in earlier stages of the diabetic eye. VEGF is expressed in retinal astrocytes, inflammatory cells, Muller and ganglion cells, RPECs, and ECs during DR (9). Production of VEGF could be regulated at the transcriptional and posttranscriptional levels. Under ischemia, oxidative stress, inflammation, and hyperglycemia conditions, some mechanisms including increased expression of hypoxia-inducible factor α (HIF-a), transforming growth factor-beta (TGFB1/2), signal transducer and activator of transcription 3 (STAT3), prostaglandins, prostacyclin, and thromboxane and decreasing of PEDF are able to induce VEGF expression (29-31). *In vitro* studies indicated that VEGF stimulated the production of nitric oxide and prostacyclin in vascular ECs (32). Further investigations have proved that VEGF induces the urokinase receptor (uPAR) expression, a protein contributed to cell adhesion in cultured retinal ECs through inducing transcriptional activation of beta-catenin (33). VEGF down-regulates the production of PEDF, the neurotrophic factor, by enhancing the matrix metallopeptidase (MMP) activity, which degrades and inactivates PEDF. PEDF has also been shown to inhibit vascular permeability induced by ocular injections of VEGF (34). VEGF has been demonstrated to increase ECs expression of ICAM-1, mitogen-activated protein kinase (MAPK), MMP, prostaglandin I2 (PGI2), and phosphatidylinositol 3-kinase /protein kinase B (PI3K/AKT). By these mechanisms, VEGF is thought to cause a breakdown of the BRB and inducing of EC growth and proliferation, angiogenesis, vascular permeability, leukostasis, and apoptosis in the retina tissue (32, 34-36). In diabetic rat models, retinal angiogenesis happened at about six months, and in this duration, VEGF was markedly overexpressed in retinal tissue and serum. Alteration of the VEGF level in serum was significantly similar to those in the retina and vitreous during the development of DR (37, 38). Furthermore, when bevacizumab, an angiogenesis blocker, was injected into the vitreous body of PDR patients, the releasing of VEGF in the serum/plasma, aqueous and vitreous was markedly abolished (39). Therefore, VEGF can be considered as a biomarker for assessing the progression of DR and the therapeutic efficiency of different agents.

***miRNAs in glucose metabolism***

**Figure 1:**
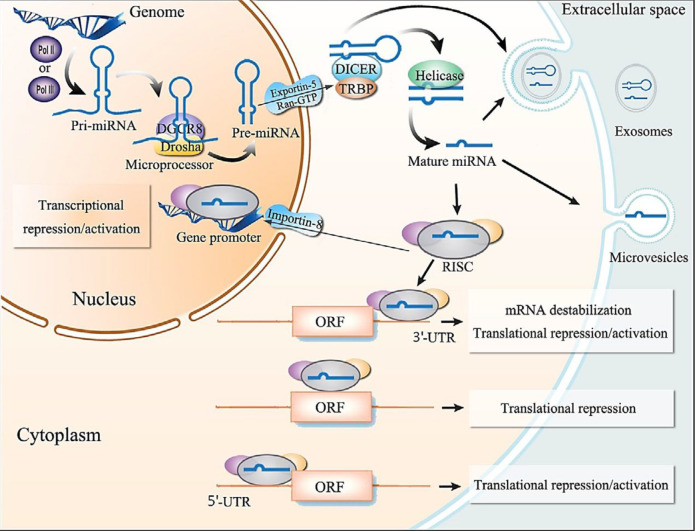
miRNA biosynthesis, function, and transportation. miRNA genes are transcribed into pri-miRNAs. The pri-miRNAs are then cleaved into the pre-miRNAs by the Drosha/DGCR8 complex. After transportation from nuclear to the cytoplasm by the Ran/GTP/Exportin-5 complex, the pre-miRNAs are processed by Dicer to produce mature miRNAs, which are ready to regulate gene expression. DGCR8: Digeorge syndrome critical region 8

miRNAs are endogenous small non-coding RNAs of 18–25 nucleotides in length that are involved in the regulation of about 60% of human protein-coding genes (40). Synthesis of miRNA is a multi-stage procedure that is started by miRNA gene transcription, and processed by different enzymes in the nucleus and cytoplasm. miRNAs can regulate gene expression by attaching to specific regions of targets, especially 3*ʹ*-UTR. The miRNA-RNA interaction leads to inhibition of translation or degradation of the target RNAs, which results in decreasing or abolishing the related protein synthesis, Figure 1 (41-43).

The alteration of miRNA expressions during the development of diabetes indicates a close relationship between these regulatory molecules and diabetes. One of the early observations on the relationship between miRNAs and glucose metabolism was in rats with knock-downed Dicer gene, in whom the formation of pancreatic islets and differentiation of insulin-secreting beta-cells were inhibited and led to growth failure and post-natal death (44). Additionally, *miR-375* was found to be high in serum and pancreatic beta-cells of patients with type 2 diabetes mellitus (T2DM), which directly decreases the gene expression and secretion of insulin through targeting phosphoinositide-dependent kinase-1 (*PDK1*) and *myotrophin*, respectively (45, 46). 

*miR-9* was introduced as a regulator of insulin secretion from pancreatic beta-cells. This miRNA is expressed in insulin-secreting cells where it can inhibit the releasing of insulin by suppressing the expression of onecut-2 (*OC2*) transcription factor and subsequently up-regulation of granuphilin in hyperglycemia conditions (47). The regulatory role of miRNAs in insulin sensitivity has also been determined, in particular the miR-103/107 family, which were detected at high levels in the liver of obese mice where they reduced insulin sensitivity. Moreover, the up-regulation of *miR-107* led to an increase in hyperglycemia, hyperinsulinemia, and liver gluconeogenesis; however, suppressing the expression of *miR-103/miR-107* led to a decrease in blood sugar and adipose tissue in obese mice by increasing caveolin-1 as a stable insulin receptor protein (48).

***miRNAs in*** ***diabetic retinopathy***

A group of miRNAs targets specific pathways contributed to the control and progression of DR. For instance, *miR-126, miR-200b,* and *miR-31* have pivotal role in the suppression of angiogenesis in DR. However, *miR-132, miR-146, miR-155*, and *miR-21* have an important role in the chronic inflammation process, which is one of the vital factors involved in the development of DR. It was demonstrated that the expression of 17 miRNAs was altered in the retina of streptozotocin-diabetic rats, and the most changes were reported to occur in miRNAs participated in the development of olfactory onions, axon production, mitogen proteins, and calcium signaling pathways (49).

**Figure 2:**
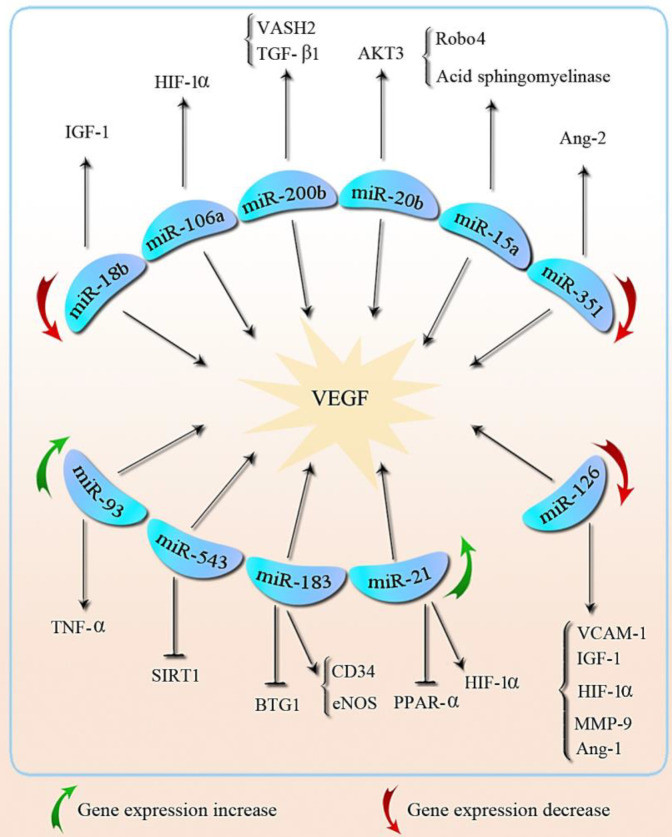
Schematic overview of the miRNAs and their effects on VEGF and other molecules contributed to the pathogenesis of diabetic retinopathy. VEGF: Vascular endothelial growth factor

A study on 300 patients with DR identified *miR-320* and *miR-27b* as angiogenesis inducers that can be used as potential biomarkers for DR patients (50). Moreover, responsive miRNAs to *VEGF*, such as* miR-17, miR-18a, miR-20a, miR-21, miR-31, *and* miR-155* are increased in retinal and vascular ECs during DR (51). *VEGF* can be regulated by AKT3, which is one of the molecules targeted by *miR-20b* during DR. Over-expression of *miR-20b* inhibited AKT3 resulted in VEGF reduction, thereby angiogenesis was inhibited (52). The relation between miRNAs and VEGF in DR has been summarized in Figure 2.

***Up-regulated miRNAs in*** ***diabetic retinopathy***

*miR-29b*

*miR-29b* as a member of the miR-29 family has been reported to be elevated in hyperinsulinemia and hyperglycemia conditions (53). Up-regulation of *miR-29b* was firstly identified in the retina of diabetic animal models, specifically after 35 days of modeling by streptozotocin injection. According to the studies, *miR-29b* and pro-apoptotic RNA-dependent protein kinase (PKR)-associated protein X (RAX), induced by *miR-29b*, are accumulated in the retinal ganglion cells and the inner nuclear layer of the retina in diabetic conditions, and they can stimulate apoptosis, a process playing a key role in the pathogenesis of DR (54, 55). In addition, Lin and colleagues observed that *miR-29b* expression is increased in RPE cells following high glucose (HG) concentrations. Moreover, attenuation of *miR-29b* has a protective effect against glucose-induced apoptosis by reducing caspase 7, phosphatase and tensin homolog (PTEN) in RPE cells (56). It was observed that *miR-29b* could act as a critical inhibitor of EMT (Epithelial-to-mesenchymal transition) processing in ARPE-19 cells by targeting neurogenic locus notch homolog protein 2 (Notch2). It was proved that RPE to mesenchymal cell transformation implicated in PDR and proliferative vitreoretinopathy (PVR) (57). Moreover, it was shown that the expression rate of miR-29b is reduced in the retina of diabetic patients with excessive angiotensinogen. The up-regulation of miR-29b prevents the increase of angiotensinogen and dramatically improves the density of vascular EC (58). Angiotensinogen, as a part of the renin-angiotensin system, participates at the beginning and progression of microcirculation injuries in various organs (59).

*miR-21*

Increment of *miR-21* was detected in the retina and ECs of diabetic mice that were mediated by nuclear factor kappa B (NF-κB) (60). Studies showed that *miR-21* inhibition greatly increased the excessive glucose-induced toxicity of ECs. On the other hand, *miR-21* Over-expression could prevent death domain-associated protein (DAXX) expression, known as a pre-apoptotic mediated factor, while removal of the *DAXX* mRNA reversed the *miR-21* suppressive effects on excessive glucose-induced apoptosis of ECs. So, miR-21 could protect ECs from apoptosis through suppressing DAXX, and its up-regulation in DR is NF-κB responsive (61). It was also shown that increased vitreous levels of miR-21 were associated with retinal fibrosis in forms of PVR and PDR. Also, miR-21 expression in the RPE cells of the ocular tissues in DR rats following HG conditions is increased and is stimulated by TGF-β, which indicates the role of miR-21 in the progression of this disease. Furthermore, miR-21 increases the proliferation and migration of ARPE-19 cells (62, 63). miR-21-3p was found to be up-regulated in the retina of diabetic mice and to have a pathogenic role by inducing the expression of HIF-1α and VEGF, and down-regulation of peroxisome proliferator-activated receptor alpha (PPARα) (64, 65). It was shown that the plasma level of *miR-21* was increased in the development of T2DM with DR; so, *miR-21* may be used as an indicator for the severity of T2DM developed to DR (66). 

*miR-34*

The miR-34 family was detected in significantly high levels in the retina of diabetic rats. In pathologic conditions, such as DR, the reproductive and migration features of RPE cells have emerged. It was revealed that *miR-34a* up-regulation could reduce RPE cell proliferation and migration through preventing its targets, c-Met, CDK2, CDK4, CDK6, p-CDC2, and leucine-rich repeat-containing G-protein coupled receptor 4 (LGR4) (60, 67, 68). *miR-34*, as a responding miRNA to P53, is up-regulated in the ocular tissue during DR. In addition, the roles of *miR-34a, b*, and *c* family have been indicated in the P53-induced apoptosis of neuromuscular and vascular ECs, aging, and cell cycle stoppage or progression in diabetic conditions (60, 69). *miR-34a* can modulate retinal endothelial cell premature aging via mitochondrial dysfunction and the inhibition of anti-oxidant activities. In this regard, Menaka *et al.*, reported that treatment of the Human REC cells with miR-34a inhibitor halted HG-induced mitochondrial dysfunction and up-regulation of aging-related factors, whereas miR-34a mimic application resulted in the loss of mitochondrial biogenesis factors (i.e., PGC-1α, NRF1, and TFAM) and the mitochondrial anti-oxidants, thioredoxin reductase 2 (TRXR2) and superoxide dismutase 2 (SOD2), and increased cellular aging (70). 

*miR-195*

Increased glucose oxidative stress accelerates the aging of endothelial and retinal cells during diabetes, but this process is inhibited by silent information regulator 1 (SIRT1). Increased expression of *miR-195* was detected in human retinal ECs (HRECs) exposed to HG, and was associated with reduced expression of SIRT1 (10, 71). Mitofusin 2 (MFN2) is another miR-195 target. MFN2 is a multi-functional mitochondrial membrane protein that protects the mitochondrial membrane against oxidative stress and diabetes complications (72). miR-195 promotes HRECs injury induced by oxidative stress through targeting MFN2 in the retina of diabetic patients and ultimately leads to the tube formation and increased permeability of the BRB (71, 73). Astragalus polysaccharides (APS) application has been shown to abolish high expression of miR-195 in RPE cells pretreated with HG. APS attenuated the mitochondrial damage, oxidative stress and cell apoptosis caused by HG in RPE cells through down-regulating *miR-195* expression. Furthermore, high expression of *miR-195* abolished the beneficial effects of APS on the HG-induced RPE cells (74). 

*Other miRNAs*

It was illustrated that abnormality in the miR-365/tissue inhibitor of metalloproteinase 3 (TIMP3) pathway is associated with the pathogenesis of Müller gliosis, and the visual impairment in DR. Increased levels of *miR-365* implicated gliosis in Müller cell via oxidative stress induction in DR rats. When *miR-365* was inhibited, TIMP3 expression was promoted, Müller cell gliosis was reduced, and retinal oxidative stress was alleviated (75).

*miR-1273g-3p* was found at high level expression in streptozotocin-induced DM RPE cells. This miRNA has a key role in DR progression by modulating the autophagy-lysosome pathway that is an intracellular self-digestive complex. Lysosomal impairment and autophagy defects are early events in the DR pathogenesis. Therefore, it was suggested that autophagy pathways could be considered as a novel therapeutic option for DR treatment. In this context, Ye and colleagues determined that *miR-1273g-3p* mimic enhanced the expression of DR-related molecules including MMP-2, MMP-9, TNF-α, ALP-related LC3, cathepsin B, and cathepsin L factors (76).

The augmentation of *miR-155* in the retina and ECs was shown in diabetic mice mediated by NF-κB. *miR-155* involves in the signaling pathways of immunity mediators. A high level of *miR-155* was detected in the blood of PDR patients that has a negative correlation with the expression of TGF-β and the number of Treg cells. It was known that the percentage of Treg cells and also TGF-β expression are usually reduced in PDR patients (77, 78).

It was determined that the plasma level of *miR-93* is positively correlated with the course of disease and the levels of fasting plasma glucose, HbA1c, TNF-α, and VEGF in T2DM patients, indicating that the plasma level of *miR-93* is a risk factor for developing DR (79). Zhen *et al.* showed that *miR-183* is overexpressed in the retinal tissues of DR rats. The *miR-183* up-regulation inhibited B-cell translocation gene 1 (*BTG1*), activated the PI3K/Akt/VEGF signaling pathway, and increased CD34, endothelial nitric oxide synthase (eNOS), and ROS. *BTG1*, an anti-proliferative gene, is a translocation partner of the *c-MYC*. It was also indicated that *miR-183* Over-expression or *BTG1* knockdown stimulated apoptosis, cell growth, and tube formation of vascular endothelial cells in DR rats (80). Qian and colleagues proved that *miR-138* is overexpressed in HG-induced ARPE-19 cells and identified *SIRT1*, an anti-apoptotic molecule, as a direct target of *miR-138*. The overexpressed *miR-138* diminished the supportive effect of salidroside (SAL), the main ingredient from *Rhodiola rosea* L, on HG-injured ARPE-19 cells. Moreover, SAL induced PI3K/AKT and 5’ AMP-activated protein kinase (AMPK) pathways and SIRT1 expression by adjusting *miR-138* (81). 

It was indicated that *miR-204-5p* is markedly up-regulated in the retina of diabetic rats. This miRNA inhibited autophagy through down-regulation of the microtubule-associated protein 1 light chain 3 (LC3B)-II. LC3B, comprising two forms (LC3B-II and I), is critical for the formation of the autophagosome. During the activation of autophagy, LC3B-I is converted to LC3B-II, which is able to enter into the autophagosome membrane (82). Levels of *miR-223-3p* have been reported to be overexpressed in DR serum samples and in HRECs during hyperglycemia. In this context, F-box and WD repeat domain-containing 7 (*FBXW7*) was identified as the target of *miR-223-3p*. *FBXW7* has been determined as a tumor suppressor, which has pivotal roles in cell proliferation, differentiation, and apoptosis (83). *miR-377* is another up-regulated miRNA in HG-induced HREC and it has been shown that its down-regulation repressed HG and hypoxia-induced inﬂammation and angiogenesis in HREC by the direct up-regulation of *SIRT1* (84). According to the findings by Ning and colleagues, *miR-543* is overexpressed in HG-induced HRECs and targets 3*ʹ*-UTR of *SIRT1* that resulted in *VEGF* down-regulation. It was found that lncRNA *SNHG7* directly prevented *miR-543* expression, which causes the up-regulation of *SIRT1* and down-regulated the *VEGF* expression in HG-induced HRECs (85). In other investigation, *SIRT1* down-regulated by *miR-23b-3p* was revealed as the main cause of HG-induced metabolic memory in HRECs. Down-regulation of *miR-23b-3p* could abolish the acetylated NF-κB expression following the SIRT1 induction, which resulted in relief of metabolic memory effects induced by HG (86).

***Down-regulated miRNAs in*** ***diabetic retinopathy***

*miR-200b*

Expression of *miR-200b* in HG-stimulated endothelial and retinal cells of streptozotocin-induced diabetic rats was decreased and this reduction was associated with the VEGF up-regulation, at protein and mRNA levels (87). It was revealed that the level of *miR-200b* in the serum of DR patients was lower than healthy individuals (88). Transfection of ECs with *miR-200b* gene and injection of this miRNA mimic into diabetic animal models reduced the expression of diabetes-induced VEGF, vascular permeability, and glucose-induced angiogenesis in the animal eyes. Furthermore, the use of miR-200b antagonists increased VEGF expression in animal models. The mechanisms regulating *miR-200b* in DR have not been well distinguished. However, it was indicated that the polycomb repressive complex 2 (PRC2) represses *miR-200b* expression through its histone H3 lysine-27 trimethylation. In this regard, the prohibition of PRC2 increases *miR-200b* while reducing VEGF (89, 90). One more study on T1DM rats showed that the expression of *miR-200b* was increased in these rats, which causes a reduction of Oxr1, as a resistance agent supporting oxidative stress conditions (91). In additional study, an increase of 4-hydroxynonena-induced apoptosis in the Müller cells was observed following transfecting with the* miR-200* gene. These controversies regarding the supportive or harmful role of *miR-200* in the retina of DR may be due to several factors such as the duration of diabetes, various diabetic animal models (genetically diabetes or streptozotocin-induced diabetes), or type of cultured cells (Müller or ECs). In addition, the *miR-200b* level in glucose-stimulated ECs was decreased along with increasing VEGF and TGF-β1. *miR-200b* inhibited the proliferation of ECs and retarded the DR progression by regulating the *VEGF* and *TGF-β1* expression (92). Yuzhi and colleagues indicated that vasohibin-2 (VASH2) involved in the promotion of angiogenesis in tumor tissues is regulated post-transcriptionally by *miR-200b/c* in vascular endothelial cells of epiretinal fibrovascular membranes from PDR patients. VASH2 was also observed to inhibit cell migration and proliferation from Day 2 to Day 4 (93).

*miR-146*

Lower expression of *miR-146a* was detected in glucose-stimulated ocular ECs of rats with T1DM. This miRNA was shown to control the expression of the fibronectin gene (94). One of the main deficiencies associated with excess glucose is the production of fibronectin, an extracellular protein that is overexpressed in DR. Consequently, down-regulation of *miR-146a* can be the main mechanism for increasing this extracellular matrix protein and the use of *miR-146a* mimic led to a decrease in fibronectin levels during DR (95).

It was shown that *miR-146a* up-regulation has a negative effect on the interleukin-1 receptor (IL-1R), toll-like receptors (TLRs), and NF-κB activation through targeting tumor necrosis factor receptor-associated factor 6 (*TRAF6*) and also interleukin-1 receptor-associated kinase 1 *(IRAK1) *(96). Cowan and colleagues observed a negative regulatory mechanism of *miR-146a* on thrombin-induced G-protein–coupled receptor (GPCR)-mediated NF-κB activation by targeting caspase recruitment domain family member 10 (*CARD10*) in HRECs (97). *miR-146a* Over-expression decreased TNF-α and TLR4/NF-κB expression levels and abolished MyD88-dependent and non-dependent pathways in the ocular ECs in hyperglycemia condition (96). In addition, *miR-146a* reduced the expression of ICAM responding to NF-κB in ocular ECs by targeting *IRAK1* (98). *miR-146a* and IRAK1 exhibit daily fluctuations in the anti-phase. This rhythmic pattern is disrupted during diabetes, leading to an increase in the expression of *ICAM*, *VEGF*, and *IL-1β*, and ultimately the development of DR. Also, down-regulation of *CARD10, IRAK1, *and* TRAF6* was observed following the intravitreal injection of lenti-miR-146a (99). 

Recent researches showed that *miR-146a* is down-regulated in glucose-stimulated ocular ECs, and its Over-expression has led to the suppression of *STAT3* and *VEGF* by reducing IL-6 in these cells (95). In DR patients, *miR-146b-3p* Over-expression resulted in the inhibition of the expression and activity of adenosine deaminase 2 (ADA2). As the anti-inflammatory effects of adenosine were proven, it was demonstrated that *miR-146b-3p* exhibits anti-inflammatory effects through ADA2 inhibition in the ocular tissue during diabetes (100). It was found that amadori-glycated albumin (AGA) treatment of monocytes/macrophages increased ADA2 expression, ADA2 activity, and TNF-α release. Transfection of macrophages with *miR-146b-3p* reversed changes in AGA-stimulated cells. Also, *miR-146b-3p* modified the pattern of disrupted ZO-1 and decreased leukocyte adhesion to HRECs (101). Moreover, it is reported that *miR-146a-5p* was down-regulated in hyperglycemia and/or hypoxia RPE cells. The up-regulation of this miRNA reversed decreased cell viability, enhanced permeability, and cell migration under DR conditions. Also, luciferase assays indicated that *miR-146a-5p* targeted *HIF-1α* and roundabout 4 (*Robo4*) directly (102).

*miR-126*

Retinal neoangiogenesis, as a key pathologic change in PDR, is associated with significant visual loss, which is mediated by various angiogenic factors including VEGF, insulin/IFG, and HIF-1α. *miR-126* has a vital role in the DR pathogenesis through regulating *VCAM-1*, *VEGF*, and *Ang-1* expression. It was demonstrated that additional deletion of *miR-126* in mice causes leaky vessels and hemorrhaging, as a result of vascular integrity destruction (103). Over-expression of *miR-126* reduced VEGF, IGF, and HIF-1α and finally suppressed angiogenesis in the retina under ischemic conditions. Regulation of angiogenic factors by *miR-126* may depend on p38, extracellular signal-regulated kinases (ERK), and MAPK pathways. During retinal ischemia, *miR-126* down-regulation may stimulate p38 and ERK signaling pathways and subsequently, augment the expression of angiogenic factors (104). Ye and colleagues showed that the *miR-126* expression was attenuated under hypoxia conditions in RF/6A cells and the retina of streptozotocin-induced diabetic rats. The revival of *miR-126* expression led to the inhibition of angiogenesis induced by hypoxia, via inhibiting *VEGF*, *MMP-9*, and cell cycle (105). In addition, it was indicated that the expression of *miR-126* in human retinal pericytes is reduced under HG conditions. Extracellular vesicles (EVs) derived from mesenchymal stem cells (MSCs) that were preserved in similar conditions of diabetes were involved in vascular instability and increased angiogenesis through paracrine processes. Many studies reported that the expression of VEGF and HIF-1α increased under hypoxia conditions (106, 107). In MSCs derived from angiogenesis under hypoxic/hyperglycemic conditions, the expression of *miR-126* reduced in pericytic cells that resulted the expression of angiogenic factors like HIF-1α and VEGF (108). Also, Rezk and colleagues found low level of serum *miR-126* in diabetic patients with known complications (particularly major vascular complications and retinopathies) compared to those without obvious complications (109). 

*miR-15 and* *miR-16*

Under the hyperglycemia condition, the expression of *miR-15b/16*, as two important miRNAs in DR, was decreased in HRECs. Also, the up-regulation of *miR-15b* and *miR-16* down-regulates suppressor of cytokine signaling 3 (*SOCS3*) and *TNF-α*, while the level of IGF binding protein-3 (IGFBP-3) and phosphorylation of the insulin receptor (IR) Tyr1150/1151 were increased. These findings indicate the important role of *miR-15b/16* in the suppression of insulin resistance, leading to HREC protection from apoptosis induced by hyperglycemia (110). The insulin receptor substrate-1* (IRS-1)* bioinformatically and experimentally is a downstream target for *miR-15b*. Liu and coworkers showed that *miR-15b* was lowly expressed in the DR rat model, whereas IRS-1 was highly expressed in EC and RP cells. After Over-expression of *miR-15b*, viabilities of EC and RP cells were reduced and β-catenin expression was prevented. It was demonstrated that *miR-15b *regulates *IRS-1* via Wnt/β-catenin signaling pathway (111).

Ye and colleagues demonstrated the inhibitory roles of *miR-15a *and* miR-16* on the pro-inflammatory signaling pathway and retinal leukostasis in DR. Hyperglycemia attenuates expression of *miR-15a *and* miR-16*, while Over-expression of these two miRNAs significantly reduces pro-inflammatory factors such as TNF-α, IL-1β, and NF-κB in HRECs under HG situation. It was also proved that the absence of these two miRNAs increased retinal leukostasis, IL-1β, CD45, TNF-α, and NF-κB. Thus, *miR-15a *and* miR-16* play an important role in the reduction of retinal leukostasis by suppressing inflammatory pathways (112). *miR-15a *and* b* are reduced in the retina of diabetic patients and this inhibition is associated with vascular repair insufficiency due to increased expression of acid sphingomyelinase, pro-inflammatory cytokines, and VEGFA molecules in the RPE and ECs (113-115). The role of *miR-15a *in the regulation of Robo4 was indicated by Gong and colleagues. VEGF and Robo4 are co-expressed in the fibrovascular membrane from PDR patients. In the late stage of DR, Robo4 and VEGF worked together to intensify DR development. *miR-15a* could down-regulate Robo4 and VEGF to ameliorate DR progress (116). 

*miR-125*

*miR-125* has been shown to be down-regulated in HG-induced human retinal microvascular endothelial cells (HRMECs) resulting in an increase in vascular endothelial-cadherin (VE-cadherin) and subsequently angiogenesis in DR condition. VE-cadherin is a cell adhesion molecule positioned at the endothelial junction and has a pivotal role in neovascularization and vascular permeability. It was investigated that lncRNA* MALAT1* up-regulation is responsible for the *miR-125* decrease. In this regard, the knockdown of *MALAT1* attenuated the migration, tube formation, proliferation and vascular permeability of HG-induced HRMECs by up-regulating *miR-125b* (117, 118). Gong and colleagues indicated that *miR-125b-5p* were decreased under hyperglycemia and/or hypoxia condition in RPE cells that was accompanied with decreased cell viability and promoted permeability, cell migration, and the expression of HIF-1α, SP1, and Robo4. The up-regulation of *miR-125b-5p* reversed these changes and gene expression patterns. Also, luciferase assays demonstrated that *miR-125b-5p* targeted *SP1* and* Robo4* (102). Robo4 is presented in vascular endothelial cells and plays an important role in pathological neoangiogenesis and the maintenance of blood vascular stability. SP1 involved in tumor progression and cell adaption for hypoxia. Furthermore, SP1 is vital for the basal expression of Robo4 in vascular endothelial cells (119). 

*Let-7*

The let-7 family is a group of miRNAs that have important roles in tissue differentiation and tumor suppression (120). *Let-7* was demonstrated to represses pathological ocular angiogenesis in DR. Let-7 family is expressed in endothelial, retinal and ARPE-19 cells (121). Over-expression of *let-7* in animal models was associated with the appearance of NPDR features, including retinal vessels and pericytes defects; however, does not advance to PDR. Also, *let-7* Over-expression can suppress the proliferation, networking and migration of ECs; in contrast, *let-7* suppression has an opposite effect. Besides, it was observed that let-7 transgenic mice, after laser-induced damage, showed a lower level of neovascularization in the choroid, while the use of anti-let-7 significantly increased the neovascularization of the choroid. Therefore, although *let-7* plays a role in the NDPR process, it inhibits angiogenesis and neovascularization in the choroid (122).

*miR-145*

In a study, it was observed that *miR-145* was down-regulated in HG-induced ARPE-19 cells and HRMECs. The interaction between baicalin (BAI), a flavonoid extracted from *Scutellaria baicalensis*, and *miR-145* showed that this flavonoid could promote *miR-145* expression. More importantly, *miR-145* repression returned the protective impacts of BAI on HG-treated ARPE-19 cells. Additionally, it was observed that BAI inhibited the activation of NF-κB and p38MAPK signaling pathways by up-regulating *miR-145* that resulted in suppressing apoptosis and inflammation (123). However, the expression of *miR-145* significantly up-regulated in the retinal ganglion cells (RGCs) incubated at HG. Zhang and colleagues showed that down-regulation of *miR-145* abolished cell proliferation capacity and cell apoptosis in HG-induced RGCs. They found that *FGF5* is a target for *miR-145* and *FGF5* knockdown decreased the protective effects of *miR-145* down-regulation (124). It seems that the role of *miR-145* varies in different types of retinal cells.

*Other miRNAs*

Ang-2 and VEGF are involved in regulating vascular formation and has been found to be controlled by *miR-351*. The interaction between *miR-351*, Ang-2, and VEGF plays an important role in vascular responses to hypoxia. Hypoxia conditions in both *in vitro* and *in vivo* models for DR results in a significant reduction in *miR-351* level, whereas VEGF and Ang-2 expression is significantly up-regulated, suggesting the protective effect of the *miR-351* up-regulation (125). Increased expression of *miR-106a* significantly reduced the levels of HIF-1α and VEGF that prevented HG-induced permeability in T1DM in an animal model. Since VEGF and HIF-1α play essential roles in DR progression and other ocular diseases, such communications may be used in therapeutic strategies (126).

*miR-18b* has been shown to reduce the VEGF expression and HREC proliferation stimulated with HG through inhibition of IGF-1/IGF1R signaling pathway. Wu and colleagues showed that the down-regulation of *miR-18b* in HRECs stimulated with HG increased cell proliferation and VEGF production. It was revealed that *miR-18b*, directly by targeting *IGF-1* and inducing *IGF-1,* prohibits the beneficial effects of *miR-18b*. A similar study demonstrated that the prevention of *miR-27a* enhanced the Bax protein, IL-6, IL-1β, TNF-α, and TLR4 expression and caspase-3/9 activity in HG-treated RPE cells (127). Previous reports have postulated the role of *miR-150-5p* in the inhibiting of angiogenic factors *in vitro*. Also, its down-regulation has been shown during pathological neovascularization in oxygen-induced proliferative retinopathy of mice (128). Chen and colleagues indicated that *miR-455-5p* expression was decreased in ARPE-19 cells stimulated with HG. In this context, the application of *miR-455-5p* mimic significantly improved cell viability and inhibited HG-caused apoptosis. Subsequently, the up-regulation of this miRNA markedly alleviated HG-induced oxidative stress injury. Furthermore, *miR-455-5p* up-regulation remarkably attenuated HG-stimulated inflammatory response through suppressing the inflammatory cytokines like IL-6, IL-1β, and TNF-α release in ARPE-19 cells. Besides, *miR-455-5p* negatively regulated the *SOCS3* expression. It should be mentioned that the SOCS3 restoration diminished the helpful effects of *miR-455-5p* on the cell apoptosis (129). 

Dong and coworkers showed that *miR-30a* expression is down-regulated in the retina of diabetic rats. The researchers demonstrated that *miR-30a* is involved in the inhibition of pro-inflammatory cytokines releasing by targeting *NLRP3*. Mechanistically, S100A12 Over-expression was found to be the cause of *miR-30a* down-regulation in the retina of the DR rat (130). S100A12 comprises the largest group of calcium-binding proteins, secreted from activated neutrophils and macrophages, and it has been confirmed that they have significant roles in exacerbating inflammatory response (131). *miR-1470* is another down-regulated miRNA in the serum of DR patients and HRECs under hyperglycemic conditions. Epidermal growth factor receptor* (EGFR)* was confirmed as the target of *miR-1470*. Moreover, it was shown that lncRNA* KCNQ1OT1* Over-expression is the cause of *miR-1470* down-regulation in HRECs under hyperglycemic conditions (132). 

***Conclusion and perspectives***

DM is a silent but dangerous disease that its prevalence is increasing worldwide and consequently the incidence of diabetes-related complications such as DR is accelerating. DR is still the first cause of blindness in westernized countries; therefore, a deep and detailed understanding of the pathophysiological mechanisms underlying the development of DR is necessary for improving the standards of care. miRNAs are novel types of gene expression regulatory factors that their contributions to etiology and pathologies of different abnormalities such as DR have been recently documented. As mentioned in this review, miRNAs regulate different pathological alterations during DR, which include cell proliferation, apoptosis, inflammation responses, microcirculation impairments, oxidative stress, and cellular death by controlling the key molecules, especially VEGF. Therefore, miRNA antagonists or mimics as a novel class of drugs could be potentially helpful to control the occurrence and progression of pathological changes during DR. Evaluation of the interaction between lncRNAs with miRNAs involved in DR pathogenesis may reveal the desired perspectives in the next generation of DR-related studies. There are complex outcomes in our basic and clinical knowledge, which prevents the finding of better therapeutic and diagnostic approaches for diseases such as DR; this and other review articles may help to achieve further clinical benefits. 

**Table 1:** Summary of microRNAs and their target molecules in diabetic retinopathy pathogenesis

**miRNA**	**up/down regulation**	**targets**	**effects**		**reference**
**miR-195**	upregulated	downregulation of SIRT1 and MTF2	induction of oxidative stress		(71, 73)
**miR-29b**	upregulated	upregulation of PTEN, caspase-7, and RAX	induction of apoptosis		(55, 56, 58)
**miR-21**	upregulated	induction of HIF-1α, VEGF expression and downregulation of PPARα	induction of proliferation and migration of RPE cells and retinal fibrosis		(61)
**Let-7**	downregulated	induction of the HMGA2 expression	induction of angiogenic behaviors		(121, 122)
**miR-146a**	downregulated	induction of fibronectin, TLR, IL-1R, NF-κB, TNF-α and ICAM expression	inducing of the inflammatory response	(94, 97, 99)	
**miR-200b**	downregulated	induction of VEGF, VASH2, and TGF-β1 expression	induction of microvascular abnormalities	(88, 91, 92)	
**miR-15b** **and miR-16**	downregulated	upregulation of TNF-α, SOCS3, IL-1β, NF-κB, IGFBP 3 and phosphorylation of insulin tyrosine receptor	induction of the inflammatory response and improving insulin sensitivity	(110, 111)	
**miR-15a** **and miR-16**	downregulated	upregulation of Robo4, VEGF, acid sphingomyelinase, CD45, IL-1β, TNF-α, and NF-κB	induction of the inflammatory response, retinal leukostasis and DR development	(121)	
**miR-126**	downregulated	upregulation of VCAM-1, VEGF, IGF, HIF-1α, MMP-9, and Ang-1	induction of the inflammatory response and improving microcirculation	(105, 109)	
**miR-93 **	upregulated	upregulation of TNF-α and VEGF	induction of inflammation and microvascular abnormalities	(79)	
**miR-351**	downregulated	upregulation of Ang-2 and VEGF	induction of microcirculation impairment	(125)	
**miR-155 **	upregulated	downregulation of TGF-β and reducing the number of Treg cells	insufficiency of the immune response	(78)	
**miR-23b-3p**	upregulated	downregulation of SIRT1	induction of metabolic memory	(86)	
**miR-365**	upregulated	causes Müller cell gliosis and retinal oxidative stress via reduction of Timp-3	induction of oxidative stress and gliosis	(75)	
**miR-1273**	upregulated	upregulation of MMP-2, MMP-9, TNF-a, ALP-related LC3, cathepsin B, and cathepsin L factors	disturbing autophagia-lysosomal digestion and Induction of metabolic memory	(76)	
**miR-34a **	upregulated	downregulation of c-Met, CDK2, CDK4, CDK6, E2F1 and p-Cdc2 and LGR4	reduction of proliferation, and migration of RPE cells	(67, 68)	
**miR-20b **	downregulated	induction of AKT3 and VEGF	induction of vascular injuries	(52)	
**miR-18b **	downregulated	upregulation of IGF-1 and VEGF expression	induction of vascular injuries	(133)	
**miR-106a **	downregulated	upregulation of HIF-1α and VEGF	induction of vascular injuries	(126)	
**miR-183**	upregulated	inhibition of BTG1, activation of the PI3K/Akt/VEGF, increase of CD34, eNOS	induction of apoptosis, cell growth and tube formation of vascular endothelial cells	(80)	
**miR-138**	upregulated	downregulation of PI3K/AKT and AMPK pathways and SIRT1 expression	induction of apoptosis	(81)	
**miR‑204**	upregulated	downregulation of LC3B-II	inhibition of autophagy	(82)	
**miR‑223**	upregulated	downregulation of FBXW7	induction of cellular proliferation and migration	(83)	
**miR-377**	upregulated	downregulation of SIRT	Induction of inﬂammation and angiogenesis	(84)	
**miR-543**	upregulated	downregulation of SIRT and upregulation of VEGF	induction of cellular proliferation and angiogenesis	(85)	
**miR-125**	downregulated	upregulation of VE-cadherin, HIF-1α, SP1 and Robo4	induction angiogenesis and permeability	(117)	
**miR-145**	downregulated	upregulation of NF-κB and p38 MAPK and Induction of FGF5	induction apoptosis and inflammation	(124)	
**miR‑27a**	downregulated	upregulation of Bax protein, IL-6, IL-1β, TNF-α and TLR4 expression and caspase-3/9	induction of inﬂammation and apoptosis	(127)	
**miR-150-5p**	downregulated	upregulation of angiogenic factors	induction OF pathological neovascularization	(128)	
**miR‐455‐5p**	downregulated	upregulation of Bax/Bcl‐2 ratio, cleaved caspase, ROS, MDA, NADPH oxidase 4. IL‐1β, IL‐6, TNF‐α and SOCS3	decrease cell viability and Induction of apoptosis, oxidative stress and inflammatory response	(129)	
**miR-30a**	downregulated	upregulation of NLRP3	induction of pro-inflammatory cytokines	(130)	
**miR‐1470**	downregulated	upregulation of EGFR	induction of cell growth and angiogenesis	(132)
